# Gastric Nodule Turned Rare Gastric Xanthoma

**DOI:** 10.7759/cureus.67570

**Published:** 2024-08-23

**Authors:** Andrej M Sodoma, James R Pellegrini, Atul Sinha, Reid Coover, Tulika Saggar

**Affiliations:** 1 Internal Medicine, South Shore University Hospital, Bay Shore, USA; 2 Internal Medicine, Nassau University Medical Center, East Meadow, USA; 3 Internal Medicine, American University of the Caribbean, Cupecoy, SXM

**Keywords:** lipid disoders, acid reflux, obesity and gerd, lipid metabolism, gastric xanthoma, esophagogastroduodenoscopy (egd), gastric nodules

## Abstract

Gastric nodules (polyps) are a common finding in routine endoscopic procedures. Uncommonly, gastric nodules turn out to be lipid-filled waxy lesions called xanthomas. In this case report, we discuss a rare incidence of a gastric nodule turning out to be a xanthoma in a 50-year-old male patient. Xanthomas of the GI tract occur by the exact mechanism as their cutaneous counterpart and are relatively uncommon. A 50-year-old male with a past medical history of gastroesophageal reflux disease (GERD), hyperlipidemia, and constipation presented to our hospital with a chief complaint of epigastric discomfort, bloating, and gastric reflux, all starting the night before admission. Gastroenterology was consulted, and they performed an esophagogastroduodenoscopy (EGD) during the admission due to the patient's age and long history of GERD. EGD showed mild gastritis and xanthelasma. The patient's upper GI symptoms improved. All other workups were negative. Right upper quadrant (RUQ) US performed showed hepatic steatosis. With lifestyle changes, the patient's alkaline phosphatase returned to normal. After a month of medical management, GERD symptoms reoccurred. Repeat EGD was performed, and xanthomatous aggregates were shown, suggesting xanthoma. Upper GI symptoms continued. Gastric xanthomas are a rare occurrence. Most xanthomas in the upper GI tract are located in the stomach and have various causes. The risk for malignancy is low; these lesions are commonly confused for malignancies, prompting biopsy and histology. Overall, xanthomas are rare findings with a sinister look but benign results.

## Introduction

Gastric nodules (polyps) are common in routine endoscopic procedures. These nodules are typically benign findings on the stomach's mucosa; however, they can rarely be a sign of inflammation and, therefore, a sign of underlying disease such as dysplasia, metaplasia, and potentially cancer [1]. Uncommonly, gastric nodules turn out to be lipid-filled waxy lesions called xanthomas.

Xanthomas are defined as localized lipid deposits within an organ system. Often, they appear due to systemic inflammation, reflecting underlying disease. The first report of xanthomas was by McFarland and McConel, who found xanthomas of the skin and described them as plaques composed of adipose tissue of benign growth [2]. The existence of gastric xanthomas, on the other hand, was discovered later, in 1929, by Lubarsch and Borchardt, who described them as gastric islands [3]. 

Xanthomas of the GI tract occur by the same mechanism as their cutaneous counterpart and are relatively uncommon. They are associated with hyperlipidemia types I/IV/V, insulin resistance, atherosclerotic coronary artery disease, biliary cirrhosis, diabetes mellitus, chronic gastritis, and H. pylori [4]. Xanthomas typically reflect an imbalance in the underlying metabolic state of an individual and should lead to further suspicion from the clinician [5]. In this case report, we discuss a rare incidence of a gastric nodule turning out to be a xanthoma in a 50-year-old male patient. 

## Case presentation

A 50-year-old male with a past medical history of gastroesophageal reflux disease (GERD), hyperlipidemia (HLD), and constipation presented to our hospital with a chief complaint of epigastric discomfort, bloating, regurgitation with non-bloody, non-billious vomiting, and gastric reflux, all starting last night. While in the ED, they performed labs and found elevated liver enzymes (Table 1).

**Table 1:** Liver Enzymes Elevated liver enzymes from the hepatic panel, from admission. AST: aspartate aminotransferase, ALT: alanine aminotransferase, Alk Phos: alkaline phosphatase

Liver Enzymes	Results (U/L)	References Ranges (U/L)
AST	96	8-48
ALT	92	7-55
Alk Phos	192	44-147

Gastroenterology was consulted, and due to the patient's age and long history of GERD, they performed an esophagogastroduodenoscopy (EGD) during the admission. They started the patient on ranitidine 300 BID and psyllium fiber 51.7% and planned for an EGD. They performed three biopsies during the EGD: antrum, body, and a 2mm gastric nodule. The EGD showed mild gastritis and xanthelasma. The patient was discharged after a few weeks and told to follow up with the Gastroenterology (GI) clinic. 

**Figure 1:**
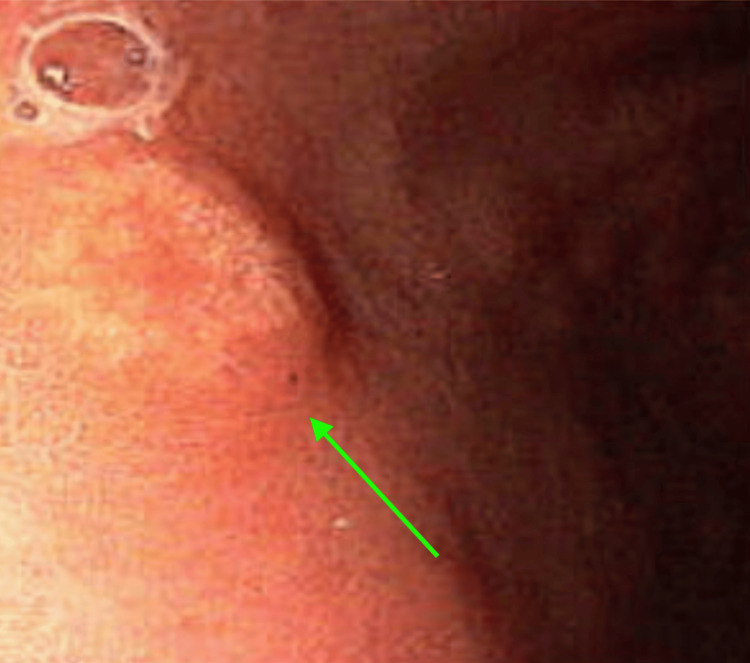
Image of gastric polyp from esophagogastroduodenoscopy (EGD) performed on initial presentation.

The patient's upper GI symptoms improved on follow-up in the GI clinic. However, they still had constipation and weight loss, with liver enzymes (Table 2). A viral hepatic panel was performed and found negative for hepatitis A, B, and C viruses and intact immunity to the hepatitis B virus. An outpatient colonoscopy was performed, and it showed grade I internal hemorrhoids. The patient had continued elevated alkaline phosphatase. Right upper quadrant (RUQ) US performed showed hepatic steatosis. With lifestyle changes, the patient's alkaline phosphatase returned to normal. 

**Table 2:** Liver Enzymes Liver enzymes from the hepatic panel performed at the GI clinic follow-up. AST: aspartate aminotransferase, ALT: alanine aminotransferase, Alk Phos: alkaline phosphatase

Liver Enzymes	Results (U/L)	References Ranges (U/L)
AST	44	8-48
ALT	41	7-55
Alk Phos	184	44-147

**Figure 2:**
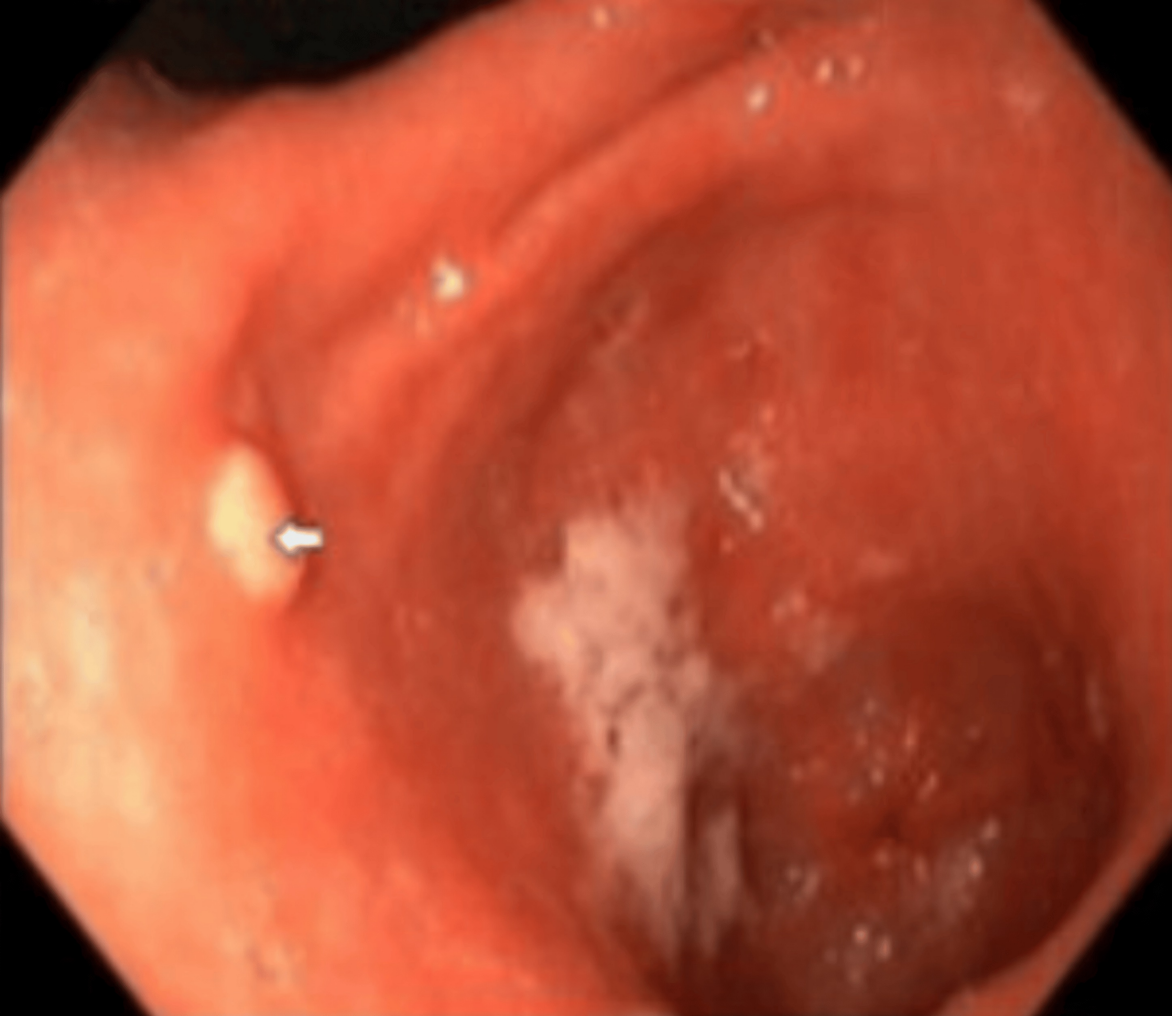
Gastric xanthoma seen on repeat esophagogastroduodenoscopy (EGD) after reoccurrence of upper GI symptoms

The patient continued to have bloating, constipation, and acid reflux for two months and returned to the GI clinic. The patient tried medical and lifestyle management, and symptoms improved. Then, a month after medical management was started, the patient experienced food regurgitation and abdominal pain. Omeprazole 10 mg twice a day was started with no improvement. Repeat EGD was performed, and xanthomatous aggregates were shown, suggesting xanthoma. Also, the results of gastric incisura reactive change, intestinal metaplasia, gastric body oxyntic mucosa, and changes suggestive of the proton-pump inhibitor (PPI) therapy effect were shown (Figure 2). 

The patient continued to have upper GI symptoms and a repeat EGD was performed. The EGD showed normal gastric fundus, the lesser and greater gastric bodies showed no significant changes, gastric incisura showed mild chronic inactive gastritis with complete intestinal metaplasia without evidence of dysplasia, the greater antrum showed focal complete intestinal metaplasia without dysplasia and a normal lesser antrum. After multiple EGDs, months of medical therapy, and numerous follow-ups, the patient is followed for gastric intestinal metaplasia.

## Discussion

Gastric xanthomas are rare. This patient was found to have them on multiple EGDs. The most common demographic is 60-year-olds (53% between the ages of 40 and 60), with a male predominance of 3:1 [5,6]. They have an estimated incidence of 0.02 - 0.8%, but autopsy studies have reported higher rates of 1.9% and 58%, which suggests that they are benign findings on endoscopy [5,6]. Few cases have been reported, and even less research has been performed on this type of lesion [7,8]. Most xanthomas in the upper GI tract are located in the stomach. The causes have been related to H. pylori infections, chronic gastritis, diabetes mellitus, and hyperlipidemia [4]. 

Although the risk for malignancy is low, these lesions are commonly confused for malignancies, prompting biopsy and histology. Four cases of cancer associated with xanthogranuloma were reported [9-11]. They found that the cancer cells neighboring the xanthoma caused a reactive proliferation of the xanthoma cells through an autocrine mechanism by paracrine and juxtacrine signaling. Also, histologically, these cells did not show atypia or mitotic figures characteristic of a malignancy [11]. The patient in this study had chronic upper GI symptoms and hepatic steatosis. 

Given the cancerous appearance of gastric xanthomas, a biopsy is warranted. Although the likelihood of a xanthoma being cancerous is low, it should still be performed. The diagnosis is also linked to disorders of lipid metabolism, which should prompt further workup if the underlying cause is not already known. In this case, on multiple EGDs, a xanthoma/xanthelasma was found, prompting a biopsy, and the result was benign every time. 

The treatment of xanthomas is through the treatment of the underlying disease. Surgical removal has not been found to benefit the patient. Surveillance or screening testing by monitoring low-density lipoprotein (LDL), high-density lipoprotein (HDL), cholesterol, and triglyceride has not been found to help predict xanthomas. 

This study has potential limitations. It is a case report, so it is an observation, making it subject to biases and confounding that could influence the production of the paper. 

## Conclusions

Xanthomas are rare, resulting from imbalances in an underlying metabolic state. They appear cancerous and, in rare cases, can grow, prompting concerns for malignancy and requiring biopsy. No lab tests have been shown to predict them, and surgical removal for the sake of treatment is futile. Treatment of the underlying disease is recommended.
